# Pulegone and Eugenol Oral Supplementation in Laboratory Animals: Results from Acute and Chronic Studies

**DOI:** 10.3390/biomedicines10102595

**Published:** 2022-10-17

**Authors:** Carla M. Ribeiro-Silva, Ana I. Faustino-Rocha, Rui M. Gil da Costa, Rui Medeiros, Maria J. Pires, Isabel Gaivão, Adelina Gama, Maria J. Neuparth, Joana V. Barbosa, Francisco Peixoto, Fernão D. Magalhães, Margarida M. S. M. Bastos, Paula A. Oliveira

**Affiliations:** 1 School of Life and Environmental Sciences (ECVA), University of Trás-os-Montes and Alto Douro (UTAD), 5000-801 Vila Real, Portugal; 2 Centre for the Research and Technology of Agro-Environmental and Biological Sciences (CITAB) Inov4Agro, 5000-801 Vila Real, Portugal; 3 Department of Zootechnics, School of Sciences and Technology (ECT), University of Évora, 7002-554 Évora, Portugal; 4 Comprehensive Health Research Centre (CHRC), 7000-811 Évora, Portugal; 5 Molecular Oncology and Viral Pathology Group, Research Center of IPO Porto (CI-IPOP)/RISE@CI-IPOP (Health Research Network), Portuguese Oncology Institute of Porto (IPO Porto), Porto Comprehensive Cancer Center (Porto.CCC), 4200-072 Porto, Portugal; 6 Laboratory for Process Engineering, Environment, Biotechnology and Energy (LEPABE), Faculty of Engineering, University of Porto (FEUP), 4200-465 Porto, Portugal; 7 Associate Laboratory in Chemical Engineering (ALiCE), FEUP, 4200-465 Porto, Portugal; 8 Postgraduate Programme in Adult Health (PPGSAD), Department of Morphology, Federal University of Maranhão (UFMA), UFMA University Hospital (HUUFMA), São Luís 65020-070, Brazil; 9 Research Department of the Portuguese League against Cancer Regional Nucleus of the North (LPCC-NRN), 4200-177 Porto, Portugal; 10 Faculty of Medicine, University of Porto, 4200-319 Porto, Portugal; 11 Institute of Biomedical Sciences Abel Salazar (ICBAS), 4050-313 Porto, Portugal; 12 Biomedical Research Center (CEBIMED), Faculty of Health Sciences of Fernando Pessoa University (UFP), 4249-004 Porto, Portugal; 13 Department of Veterinary Sciences, UTAD, 5000-801 Vila Real, Portugal; 14 Animal and Veterinary Research Centre (CECAV), Associate Laboratory for Animal and Veterinary Science—AL4AnimalS, 5000-801 Vila Real, Portugal; 15 Department of Genetics and Biotechnology, 5000-801 Vila Real, Portugal; 16 Associate Laboratory for Animal and Veterinary Sciences (AL4AnimalS), UTAD, 5000-801 Vila Real, Portugal; 17 Research Center in Physical Activity, Health and Leisure (CIAFEL), Faculty of Sports, O.Porto, University of Porto (FADEUP), 4200-319 Porto, Portugal; 18 Laboratory for Integrative and Translational Research in Population Health (ITR), 4050-600 Porto, Portugal; 19 Toxicology Research Unit (TOXRUN), University Institute of Health Sciences, CESPU, CRL, 4585-116 Gandra, Portugal; 20 Chemistry Center—Vila Real (CQ-VR), Biological and Environment Department, UTAD, 5000-801 Vila Real, Portugal

**Keywords:** antioxidant defenses, comet assay, essential oils, histopathology, mice, toxicity

## Abstract

Essential oils are natural compounds used by humans for scientific purposes due to their wide range of properties. Eugenol is mostly present in clove oil, while pulegone is the main constituent of pennyroyal oil. To guarantee the safe use of eugenol and pulegone for both humans and animals, this study addressed, for the first time, the effects of these compounds, at low doses (chronic toxicity) and high doses (acute toxicity), in laboratory animals. Thirty-five FVB/n female mice were randomly assigned to seven groups (n = 5): group I (control, non-additive diet); group II (2.6 mg of eugenol + 2.6 mg of pulegone); group III (5.2 mg of eugenol + 5.2 mg of pulegone); group IV (7.8 mg of eugenol + 7.8 mg of pulegone); group V (7.8 mg of eugenol); group VI (7.8 mg of pulegone); and group VII (1000 mg of eugenol + 1000 mg of pulegone). The compounds were administered in the food. Groups I to VI were integrated into the chronic toxicity study, lasting 28 days, and group VII was used in the acute toxicity study, lasting 7 days. Animals were monitored to assess their general welfare. Water and food intake, as well as body weight, were recorded. On the 29th day, all animals were euthanized by an overdose of ketamine and xylazine, and a complete necropsy was performed. Blood samples were collected directly from the heart for microhematocrit and serum analysis, as well as for comet assay. Organs were collected, weighed, and fixed in formaldehyde for further histological analysis and enzymatic assay. Eugenol and pulegone induced behavioral changes in the animals, namely in the posture, hair appearance and grooming, and in mental status. These compounds also caused a decrease in the animals’ body weight, as well as in the food and water consumption. A mortality rate of 20% was registered in the acute toxicity group. Both compounds modulated the serum levels of triglycerides and alanine aminotransferase. Eugenol and pulegone induced genetic damage in all animals. Eugenol increased the activity of the CAT enzyme. Both compounds increased the GR enzyme at the highest dose. Moreover, pulegone administered as a single compound increased the activity of the GST enzyme. Histopathological analysis revealed inflammatory infiltrates in the lungs of groups II, III, and IV. The results suggest that eugenol and pulegone may exert beneficial or harmful effects, depending on the dose, and if applied alone or in combination.

## 1. Introduction

Essential oils are complex mixtures, composed mainly of hydrocarbons (terpenes) and their oxygenated derivatives [1]. They are volatile, naturally occurring compounds formed as secondary metabolites in different organs of aromatic and medicinal plants, such as bark, flowers, leaves, roots, and seeds [2]. Generally, the essential oils are colorless, with a strong odor, and their availability and accessibility has increased their use and scientific interest [3]. Due to their extensive range of properties (aromatic, bactericidal, fungicidal, insecticidal, and viricidal) essential oils are widely used in food, cosmetics, dentistry, and medicine [3,4,5]. Moreover, they protect cereals from insects through contact toxicity and release of antifeedant and fumigant chemical compounds, and offer protection from fungi through inhibition of mycelium growth and spore germination, thereby increasing their shelf life [6,7].

Eugenol is a natural phenolic compound found in honey and in numerous plant extracts, including clove (*Syzygium aromaticum*) and magnolia flower (*Magnolia grandiflora*). Clove oil, containing 85–95% of eugenol, is the main source of this product, and it is widely used in pharmaceutical technology for the preparation of pharmaceutical formulas used in dentistry for analgesic and antiseptic purposes [8,9,10,11]. Humans are exposed daily to eugenol due to its high versatility. Indeed, it is present in cosmetics, deodorants, perfumes, and is used in moderate concentration as a flavoring agent in food products, mouthwashes, and toothpastes [12,13]. Studies on mice have shown that eugenol present in clove oil inhibits cytokines, acting as an anti-inflammatory compound [14]. It also acted as an analgesic in rats to which abdominal constrictions were induced by acetic acid administration [15], and an anticancer compound in the human lung adenocarcinoma cell line [11]. Eugenol also possesses antifungal activity against *Candida albicans* [16,17], and antibacterial properties against foodborne pathogens, such as *Listeria monocytogenes*, *Salmonella typhimurium*, and *Escherichia coli* [18]. Previous studies have also demonstrated that eugenol has antioxidant and anti-inflammatory properties [19]. Despite this, some negative effects related to the use of dental products including eugenol were reported, particularly skin irritation, ulcer formation, tissue necrosis, or, in rarer cases, anaphylactic shock [20]. Crises of asthma, rhinitis, contact dermatitis, and acute urticaria were also reported as a result of prolonged exposure to the eugenol present in perfumes [21].

Pulegone is present in essential oils from a variety of plants, such as catnip (*Nepeta cataria*) and peppermint (*Mentha piperita*), and is the main constituent of pennyroyal (*Mentha pulegium*) oil, which is used to induce menstruation and abortions [22,23,24]. Pulegone is described to be an insecticide and a pesticide, with different pharmacological properties, namely anti-bacterial, anti-fungal, anti-histaminic, anti-inflammatory, anti-nociceptive, and anti-pyretic [22,25,26,27,28,29,30,31,32,33]. It may also be used as an insect repellent; to flavor foods, beverages, and dental products; as a fragrance; and in medicines [34]. Humans are exposed to this monoterpene primarily through the ingestion of beverages or foods flavored with the essential oils of pennyroyal or peppermint. However, previous studies showed that pulegone can have adverse effects [24,35,36,37,38]. There are reports of human poisoning from the ingestion of pennyroyal oil, with ingestion of 10 mL resulting in moderate to severe toxicity (kidney and liver disorders, and coma), and ingestion of more than 15 mL resulting in death. Mild central nervous system toxicity and gastritis are associated with the ingestion of less than 10 mL of pennyroyal oil [39].

Considering the properties attributed to eugenol and pulegone, and based on their use as preservatives to transport cereals, the present study addressed, for the first time, the acute and chronic toxicological effects of dietary supplementation with these compounds on behavioral, physiological, hematological, histopathological, genotoxic, and enzymatic parameters of FVB/n female mice, in order to guarantee their safe use for both humans and animals.

## 2. Materials and Methods

### 2.1. Experimental Design and Conditions

All procedures were performed in accordance with the National (Decree-Law nº 113/2013) and European (European Directive 2010/63/EU) legislation on the protection of animals used for scientific purposes. The experimental protocol was approved by the Portuguese competent authority (*Direção Geral de Alimentação e Veterinária*—DGAV, approval nº 008961) and the Ethics Committee of UTAD.

**Figure 1:**
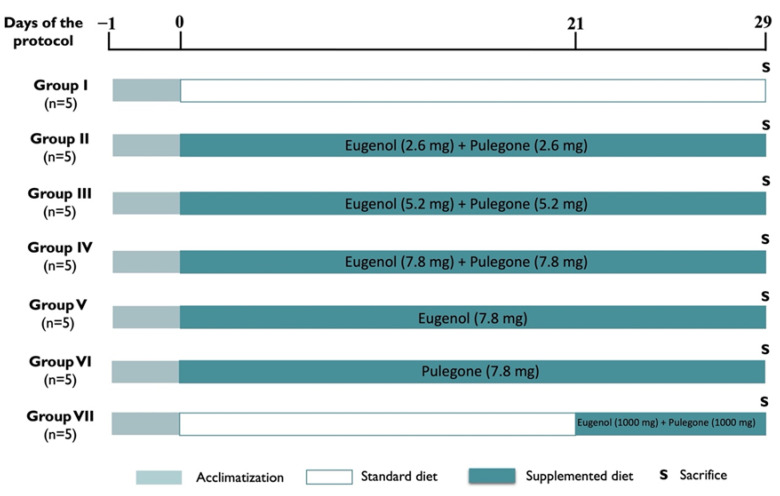
Experimental protocol, representing the chronic toxicity assay (groups II to VI) with a duration of 28 days, and the acute toxicity assay (group VII) with a duration of 7 days.

Thirty-five FVB/n female mice (*Mus musculus*) at 14–15 weeks of age were used. The animals belonged to a colony resident in the animal house of the University of Trás-os-Montes and Alto Douro (UTAD). After one week of acclimatization, the animals were randomly assigned to seven experimental groups and two experiments were performed: a chronic toxicity assay with duration of 28 days (groups II to VI) and an acute toxicity assay (group VII) with duration of 7 days (Figure 1). During the experimental protocol, the animals were kept under controlled conditions of temperature (23 ± 2 °C), humidity (50 ± 10%), air system filtration (10–20 ventilations/hour), and light:dark cycle (12-h:12-h). They were placed in hard polycarbonate cages (Eurostandard Tipo IV S 1500U, Tecniplast, Italy) using corncob for bedding (Ultragene, Santa Comba Dão, Portugal) and environmental enrichment with paper rolls. Water and food were provided *ad libitum*.

### 2.2. Diet Preparation

In a previous unpublished study, eugenol and pulegone were used to preserve 560 kg of corn in the presence of five devices, equally distributed, containing the essential oils of clove and pennyroyal, with eugenol and pulegone in equal content. After eight and a half months, the pulegone content in the corn was quantified at a concentration of 15,900 mg/kg and the same concentration was considered for eugenol. Considering the average daily intake of corn recommended for humans, the following formula suggested by Nair and Jacob [40] was used to determine the dose recommended for mice:

Animal equivalent dose (mg/kg) = Human dose (mg/kg) × Correction factor ratio

Considering the mean body weight of each mouse, as well as its average daily food intake, we determined that 32 g of corn should be incorporated *per* kg of diet, with this amount of diet representing a daily ingestion of 2.6 mg (group II) of each of these compounds per animal. Therefore, the pulegone (85%, reference P55708, Sigma-Aldrich, St. Louis, MI, USA) was incorporated into the animals’ diet at a concentration of 599 mg/kg of food, and the eugenol (99%, reference 11915000, ACROS Organics, Geel, Belgium) was incorporated at a concentration of 509 mg/kg. The eugenol and pulegone were weighed in a precision scale (Kern^®^ PLJ 750-3N, Kern & Sohn GmbH, Balingen, Germany) and stored in eppendorfs to be then incorporated in the diet. To prepare the diets, the food pellets were ground in a Bimby TM31 (Vorwerk, Wuppertal, Germany). One kilogram of diet was weighed for each experimental group and the eugenol and pulegone were added. The mixture was homogenized in a Kenwood mixer (Kenwood Chef, Japan), and the pellets were formed again, with their diameter defined by a 4 mm matrix (Eriez Magnetics, Caerphilly, UK). The double (5.2 mg, group III) and triple (7.8 mg, groups IV, V, and VI) doses were tested in the chronic toxicity study for both eugenol and pulegone. In the case of the acute toxicity assay, 1000 mg of each of the compounds were incorporated in each kg of food (group VII). A control group (group I) with a standard diet was also used (Figure 1).

### 2.3. Animals’ Monitoring

The animals were monitored daily to check their health status. The animals’ body condition, body weight, posture, hair appearance and grooming, mucosa, eyes, ears and whiskers, mental status, response to external stimuli, hydration status, respiratory and heart rate, and stool appearance were evaluated. To avoid bias, the animals were always observed by the same researcher. A score from 0 to 3 was attributed to each parameter (Table 1). The scores attributed to each parameter were summed, and if an animal reached the value of four, it was withdrawn from the study and sacrificed. According to the Canadian Council on Animal Care (CCAC), in the studies of acute toxicity the researchers focused on the primary and secondary interactions of the compound with the animal and not on the tertiary effects (anorexia) [41]. In this way, the parameter body weight was adapted for the acute toxicity assay, and a weight loss higher than 20% was not indicative for euthanasia. At the end of the experiment, an average score was determined for each group. The food and water ingestion were also monitored during the study.

### 2.4. Animals’ Sacrifice

Animals from both acute and chronic toxicity experiments were sacrificed on the 29th day of the study by an intraperitoneal injection of ketamine (Eutasil 200 mg/mL, Ceva, Algés, Portugal) and xylazine (Rompun^®^ 20 mg/mL, Bayer Healthcare S.A., Kiel, Germany), followed by exsanguination by cardiac puncture, as recommended by FELASA (Federation for Laboratory Animal Science Associations) [44]. A complete necropsy was performed, and the internal organs (heart, lungs, kidneys, spleen, and liver) were collected and weighed individually using a precision scale (KERN^®^ PLJ 750-3N, Kern & Sohn GmbH, Balingen, Germany).

### 2.5. Microhematocrit and Serum Biochemical Analysis

Blood collected directly from the heart during euthanasia was stored in heparinized tubes (Tube Lithium Heparin 0.5 mL, FL MEDICAL, Italy). Two heparinized capillary tubes per animal were centrifuged in the PrO-Vet centrifuge (Centurion, Scientific Limited) at 12,000 rotations per minute (rpm) for 5 min to determine microhematocrit value. The blood samples were then centrifuged at 1400× *g* for 15 min, and the serum was separated and frozen at −80 °C for further analysis. The concentrations of albumin, cholesterol, urea, triglycerides, and alanine aminotransferase were determined through spectrophotometric methods using an autoanalyzer (Prestige 24i, Cormay PZ), to detect potential metabolic disorders and hepatotoxic effects.

### 2.6. Comet Assay

The alkaline comet assay was performed as previously described by Collins and Azqueta [45]. Briefly, slides were precoated with low-melting-point agarose. For each animal, four slides were prepared (two for performing the assay with the repair enzyme and the other two for the assay without the enzyme). Approximately 10 μL of blood was diluted in 200 μL of ice-cold phosphate-buffered saline (PBS) in a 0.5 mL microtube to prepare a cell suspension. An aliquot of the cell suspension was placed onto the precoated slides. The slides were immersed in a lysis solution, and then electrophoresed. Following electrophoresis, the cells were immersed in PBS followed by distilled water, dehydrated in ethanol, and air-dried. The slides were then stained with 4,6-diamidino-2-phenylindole solution (Sigma-Aldrich Chemical Company, Spain) and observed using an Olympus BX41 fluorescence microscope (Tokyo, Japan) at 400×. The nucleoids were classified visually in five classes from 0 (no tail) to 4 (almost all DNA in the tail) (Collins, 2004). Fifty nucleoids were observed per mini-gel (100 per case). A genetic damage index (GDI), expressed in an arbitrary scale from 0 to 400, was obtained according to the formula:

GDI = (nucleoids class 0 × 0) + (nucleoids class 1 × 1) + (nucleoids class 2 × 2) + (nucleoids class 3 × 3) + (nucleoids class 4 × 4)

### 2.7. Oxidative Stress Parameters

At necropsy, small sections of liver were placed into 12 mL tubes containing cryopreservation medium and frozen at −20 °C for oxidative stress analysis. Then, the liver samples (approximately 0.25 g) were washed and added to 10% (*p*/*v*) tris buffer (pH 7.5). After homogenization and sonication (six pulses of 20 s with 10 s intervals), the samples were subjected to three centrifugation cycles, at 4 °C: 3000 rpm for 10 min, 14,000 rpm for 10 min, and 14,000 rpm for 15 min. The supernatant obtained in the last centrifugation was used to measure antioxidant enzymes activity: superoxide dismutase (SOD), catalase (CAT), glutathione reductase (GR), and glutathione S-transferase (GST). The total protein content of the supernatant and pellets obtained was evaluated by the Biuret method, using bovine serum albumin as standard [46].

Total SOD activity method was based on inhibition of nitroblue tetrazolium (NBT) reduction by O2−• generated by the xanthine/xanthine oxidase system, according to the method originally described by Payá [47], and the results were expressed as U·min^−1^·mg protein^−1^. CAT activity was assayed polarographically using a Clark-type oxygen electrode as described before by Del Río et al. [48], and the activity expressed in mmol H_2_O_2_·min^−1^·mg protein^−1^. GR activity was assayed as described previously by Carlberg e Mannervik (1985), and the results were expressed as min^−1^·mg protein^−1^. GST activity was evaluated, as previously described by Hatton et al. [49], and the results were expressed as µmol 1-chloro-2,4-dinitrobenzene.min^−1^·mg protein^−1^.

### 2.8. Histological Analysis

The organs collected in the necropsy were immersed in 10% neutral buffered formaldehyde for at least 24 h (ITW Reagents, Germany), and routinely processed for light microscopy. Paraffin 3-µm-thick sections were stained with hematoxylin and eosin (H&E) and observed under a light microscopy by a pathologist. The presence of inflammatory infiltrate was evaluated according to the following scores: 0—without inflammatory aggregates; 1—with less than five multifocal inflammatory aggregates; 2—with five or more inflammatory aggregates.

### 2.9. Statistical Analysis

The mean water and food consumption per animal per day, as well as relative organs’ weight were calculated according to the following formulas:

Mean food consumption per animal *per* day = (Initial food weight − Final food weight)/(Number of days between initial and final weighing × Number of animals per cage)

Mean water consumption per animal *per* day = (Initial water weight − Final water weight)/(Number of days between initial and final weighing × Number of animals per cage)

Organs’ relative weight = Organ weight/Animal body weight

For each experimental group, the ponderal weight gain (PWG) was calculated by applying the following formula:

PWG = Final body weight − Initial body weight/Final body weight × 100

At the end of the study, the mortality index was determined according to the formula:

Mortality index = (Number of animals that died during the experiment/Number of animals at the beginning of the study) × 100

Statistical analysis was performed with the GraphPad Prism 8.0 (GraphPad Software, Inc., San Diego, CA, USA). Analysis of variance (ANOVA) followed by the Tukey test was performed to compare the following variables among groups: body weight, ponderal weight gain, organs’ weight, microhematocrit, biochemical parameters, data from the comet assay, and oxidative stress parameters. A chi-squared test was performed to study the distribution of histological lesions among groups. The results are expressed as mean ± standard error (SE) and *p*-values < 0.05 were considered statistically significant.

## 3. Results

### 3.1. General Findings and Humane Endpoints

One animal from group VII (acute toxicity) died on the day of the sacrifice, some hours before it (mortality index of 20% in this group). The organs of this animal were collected and processed for histopathological analysis, but the blood samples were not collected, and the organs were not weighed. Animals from groups I (control), III (5.2 mg eugenol + 5.2 mg pulegone), and V (7.8 mg eugenol) did not show any signs of distress during the experiment, presenting a score of 0 in all weeks (Table 2). Animals from the remaining groups showed alterations in some of the parameters, mainly in the last three weeks of the experimental protocol. Groups II, IV, and VI presented a positive mean score for humane endpoints in weeks 2, 3, and 4 of the protocol. The highest mean score was registered for group VII, with a mean score of 6.8 in the last week of the experiment (Table 2). Changed body condition (score 1) was observed in four animals (80%) from group VII (1000 mg eugenol + 1000 mg pulegone) in the last week of the experiment. Body weight changes were noted in all weeks of the experiment. In the first weeks of the protocol, the body weight was changed in two animals from group IV (weight loss of 10–20% in one animal (20%) and weight loss > 20% in another animal (20%)). A weight loss between 10 and 20% was observed in one animal (20%) from group IV in week 2 and in one animal (20%) from group II in week 3 of the experiment. In the last week of the protocol, all animals (100%) from group VII exhibited a weight loss > 20%. A curved posture (score 1) was observed in weeks 2 and 3 in one animal (20%) from group IV and in one animal (20%) from group VI, and in week 4 of the experimental protocol in four animals (80%) from group VII. In the last week of the experiment, a lack of grooming (score 1) was observed in 12 animals: five animals from group IV (100%), three animals from group VI (60%), and four animals (80%) from group VII. Eyes, ears, and whiskers were changed (score 1) in one animal (20%) of group VII in the last week of the experimental protocol. Mental status was changed in the last three weeks of the experiment. Two animals (40%) from group II exhibited a changed mental status (score 1) in weeks 2 and 3 of the protocol. In the last week of the experiment, four animals (80%) from group II and three animals (60%) from group VII were inactive, and one animal from group VII (20%) was comatose (Table 3).

### 3.2. Food and Water Consumption

Except for group IV (7.8 mg eugenol + 7.8 mg pulegone), a decrease in food consumption was observed between the beginning and the end of the experimental protocol in all groups. A marked decrease in food consumption was observed in group VII (1000 mg eugenol + 1000 mg pulegone), with a mean consumption of 4.44 g in the first week and a consumption of only 0.40 g in the last week. This value was substantially lower when compared with the remaining groups, namely the control group (group I) (Table 4).

A decrease in water consumption was observed throughout the experiment in all groups, and similar to that observed for food, the group VII (1000 mg eugenol + 1000 mg pulegone) was that with the highest decrease. The data suggest that supplementation with eugenol and pulegone negatively affected the food and water consumption (Table 4).

### 3.3. Body Weight and Ponderal Weight Gain

The initial body weight was similar among groups (*p* > 0.05). Inversely, the final body weight was higher in group I (control) when compared with group VII (1000 mg eugenol + 1000 mg pulegone) (*p* = 0.000). A marked decline was observed in the body weight of the animals from the acute toxicity group (group VII) between the beginning and the end of the experimental protocol (Table 5).

The PWG of group IV (7.8 mg eugenol + 7.8 mg pulegone) was negative and statistically different from groups I (control), II (2.6 mg eugenol + 2.6 mg pulegone), and III (5.2 mg eugenol + 5.2 mg pulegone) (*p* < 0.05). The acute toxicity group (group VII) also presented a PWG negative and statistically different from group I (control) (*p* = 0.000) (Table 5). The data suggest that the supplementation with eugenol and pulegone modulates animals’ body weight and PWG when used together and in specific quantities.

### 3.4. Organs’ Weight and Relative Weight

The mean weight of the heart, lung, kidneys, spleen, and liver is presented in Table 6. The mean liver weight of animals from group III (5.2 mg eugenol + 5.2 mg pulegone) was higher and statistically different when compared with the liver weight of animals from groups I (control), II (2.6 mg eugenol + 2.6 mg pulegone), and IV (7.8 mg eugenol + 7.8 mg pulegone) (*p* < 0.05). The liver weight of animals from group VII (1000 mg eugenol + 1000 mg pulegone) was lower when compared with other groups, and statistically different from group I (control) (*p* = 0.000). The weight of the remaining organs was similar among groups (*p* > 0.05) (Table 6).

The relative weight of the heart and both kidneys was similar among groups (*p* > 0.05). The lung relative weight of the acute toxicity group (group VII; 0.010 ± 4 × 10^−4^) was higher and statistically different from group I (control; 0.007 ± 8 × 10^−4^) (*p* = 0.003). The spleen relative weight was higher in group III (0.006± 6 × 10^−4^) when compared with group IV (0.003 ± 5 × 10^−4^) (*p* = 0.005). The relative weight of liver of animals from group I (0.049 ± 1.3 × 10^−3^) was lower when compared with that from groups II (0.051 ± 3.3 × 10^−3^), III (0.055 ± 1.8 × 10^−3^), IV (0.055 ± 3.9 × 10^−3^), V (0.052 ± 2.8 × 10^−3^), and VI (0.054 ± 2.2 × 10^−3^) (*p* < 0.05). Moreover, the liver relative weight of group II was statistically different from groups III and IV (*p* < 0.05) (data not shown).

### 3.5. Microhematocrit and Biochemical Parameters

**Figure 2:**
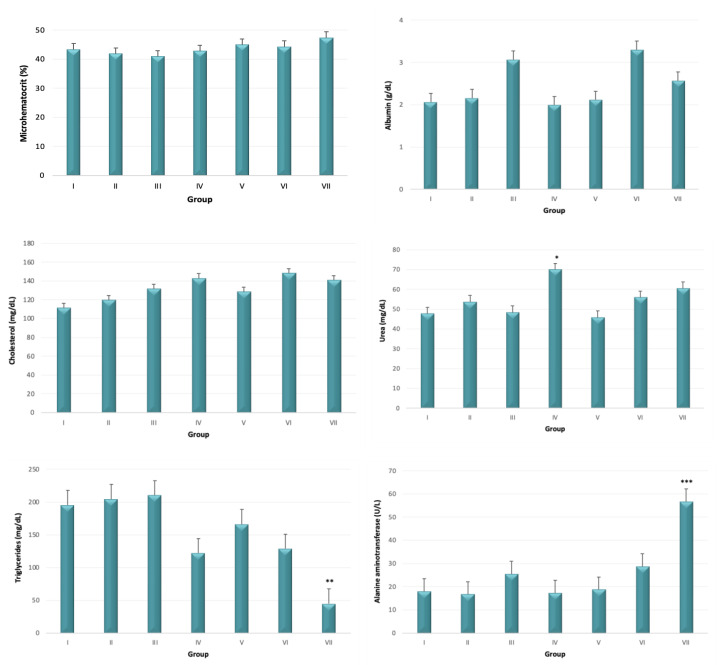
Microhematocrit (%) and serum levels of albumin (g/dL), cholesterol (mg/dL), urea (mg/d), triglycerides (mg/dL), and alanine aminotransferase (U/L) (mean ± SE) in all experimental groups: I (control); II (2.6 mg eugenol + 2.6 mg pulegone); III (5.2 mg eugenol + 5.2 mg pulegone); IV (7.8 mg eugenol + 7.8. mg pulegone); V (7.8 mg eugenol); VI (7.8 mg pulegone); group VII (1000 mg eugenol + 1000 mg pulegone). * Statistically different from group V (*p* < 0.05); ** Statistically different from groups I, II, III, and V (*p* < 0.05); *** Statistically different from all groups (*p* < 0.05).

The microhematocrit value and the serum levels of albumin and cholesterol were similar among groups (*p* > 0.05). The urea serum levels were higher in group IV (7.8 mg eugenol + 7.8. mg pulegone) when compared with group V (*p* < 0.05). Triglycerides serum levels were lower in acute exposure group (group VII) when compared with groups I (control), II (2.6 mg eugenol + 2.6 mg pulegone), III (5.2 mg eugenol + 5.2 mg pulegone), and V (7.8 mg eugenol) (*p* < 0.05), and the alanine aminotransferase serum levels were higher in the acute toxicity group (group VII), when compared with the remaining groups (*p* < 0.05) (Figure 2).

### 3.6. Comet Assay

**Figure 3:**
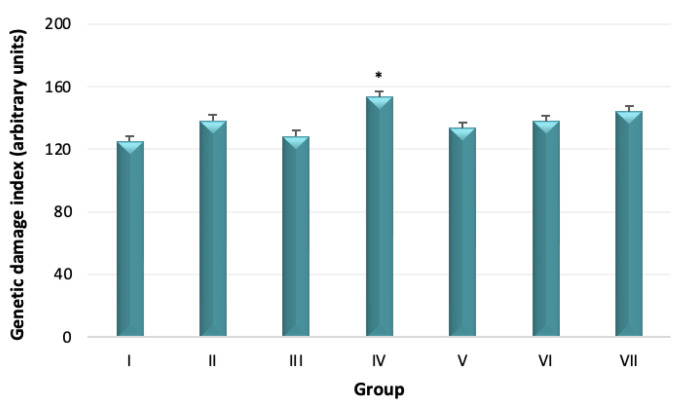
Genetic damage index (GDI, mean ± SE) in all experimental groups: I (control); II (2.6 mg eugenol + 2.6 mg pulegone); III (5.2 mg eugenol + 5.2 mg pulegone); IV (7.8 mg eugenol + 7.8 mg pulegone); V (7.8 mg eugenol); VI (7.8 mg pulegone); VII (1000 mg eugenol + 1000 mg pulegone). Counting of 100 comets per animal, in a total of four replicates per animal. * Statistically different from groups I (*p* = 0.014) and III (*p* = 0.037).

The GDI was higher in all experimental groups when compared with the control group (group I). A statistically significant difference was reached between group IV (7.8 mg eugenol + 7.8 mg pulegone) and control group (*p* = 0.014). A significant difference was also observed between group III (5.2 mg eugenol + 5.2 mg pulegone) and group IV (7.8 mg eugenol + 7.8 mg pulegone) (*p* = 0.037) (Figure 3).

### 3.7. Oxidative Stress Parameters

**Figure 4:**
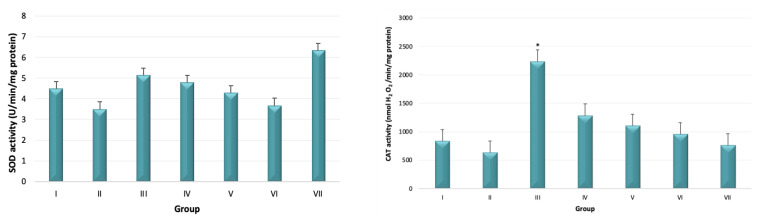

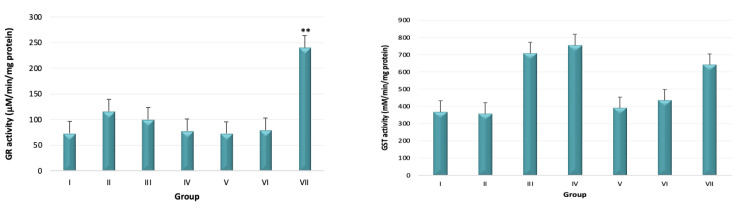
Activity of antioxidant enzymes: superoxide dismutase (SOD), catalase (CAT), glutathione reductase (GR), and glutathione S-transferase (GST), in all experimental groups: I (control); II (2.6 mg eugenol + 2.6 mg pulegone); III (5.2 mg eugenol + 5.2 mg pulegone); IV (7.8 mg eugenol + 7.8. mg pulegone); V (7.8 mg eugenol); VI (7.8 mg pulegone); VII (1000 mg eugenol + 1000 mg pulegone). * Statistically different from groups I, II, V, VI, and VII (*p* < 0.05); ** Statistically different from all groups (*p* < 0.05).

The activity of antioxidant enzymes SOD, CAT, GR, and GST were measured. The SOD activity was not statistically different among groups (*p* > 0.05). Despite this, there seems to be a decreasing trend of SOD activity with an increasing dose of eugenol and pulegone (groups II, III, and IV) (*p* > 0.05). The SOD activity was higher in animals treated with eugenol (group V) when compared with those treated with pulegone (group VI). Although the difference did not reach a statistically significant difference (*p* > 0.05), the SOD activity was higher in the acute toxicity group (group VII) when compared with the remaining groups. The CAT activity of group II (2.6 mg eugenol + 2.6 mg pulegone) was higher when compared with remaining groups, and statistically different from groups I, II, V, VI, and VII (*p* < 0.05). The GR activity was higher in the acute toxicity group (group VII) when compared with the remaining experimental groups (*p* < 0.05). The activity of GST was higher in groups treated with higher doses of eugenol and pulegone (groups III and IV) when compared with the remaining groups. However, the difference did not reach the level of statistical significance (Figure 4).

### 3.8. Histopathological Analysis

No macroscopic changes were observed. Histopathological analysis revealed the presence of inflammatory infiltrate in the lung, kidneys, and liver, with a score varying from 0 to 2. The inflammatory infiltrate in the liver and kidneys was similar among groups. Inversely, the lung inflammatory infiltrate score was higher in groups IV (7.8 mg eugenol + 7.8 mg pulegone) and V (7.8 mg eugenol), when compared with groups I (control), II (2.6 mg eugenol + 2.6 mg pulegone), and III (5.2 mg eugenol + 5.2 mg pulegone) (*p* < 0.05). Moreover, an increase in the mean inflammatory score was observed from group II to group IV, suggesting that eugenol and pulegone may cause lung damage. As the group V (7.8 mg eugenol) presented a higher mean score when compared with group VI (7.8 mg pulegone), it seems that eugenol was responsible for the damage.

**Figure 5:**
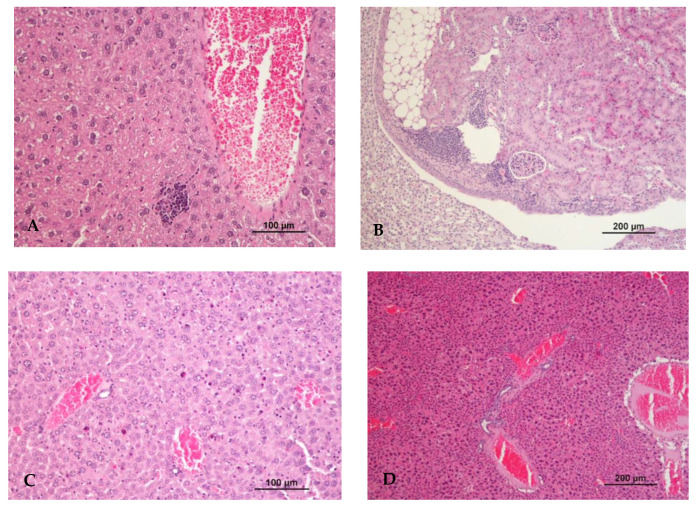
Histopathological changes observed in the liver and kidneys. (**A**) Focal inflammatory infiltrate of lymphocytes in the liver (Group IV); (**B**) multifocal inflammatory infiltrate of lymphocytes in the kidney (Group V); (**C**) liver: hepatocellular apoptosis (Group VII); (**D**) liver: marked centrilobular hepatocellular hypertrophy (Group VII). Hematoxylin and eosin staining.

In addition to the inflammatory infiltrate, multiple isolated apoptotic hepatocytes were observed in two animals (40%) from the acute toxicity group (group VII) (Table 7, Figure 5). Moreover, centrilobular hepatocellular hypertrophy and hepatocyte karyomegaly was observed in all groups. An increase in the number of animals with these histopathological changes was observed with the increase in the dose of eugenol and pulegone (between groups II (60%) and III (80%)). A higher number of animals with histopathological changes were observed in group V (80%) when compared with group VI (60%), suggesting that eugenol was responsible for the liver damage, similar to the results regarding lung damage. All animals (100%) from the acute toxicity group (group VII) presented marked centrilobular hepatocellular hypertrophy and hepatocyte karyomegaly (Table 8, Figure 5).

## 4. Discussion

Essential oils are recognized for their antibacterial, antifungal, insecticidal, and antioxidant properties, and are widely studied by the pharmaceutical and food industries to determine their best application without endangering the final consumer. In order to be used in the food industry, it is necessary to understand the effects of essential oils on the animal and human organisms [50,51,52,53].

Eugenol is the main component of clove oil [54]. Due to its redox properties, this phenolic compound plays an important role in neutralizing free radicals and breaking down peroxides [52], suggesting its anti-inflammatory properties [55]. Pulegone is one of the main components of pennyroyal oil, and causes depletion of glutathione levels and induces lesions in several organs, namely in the liver [56]. However, it is reported to have an anti-inflammatory action in RAW 264.7 cells induced by lipopolysaccharide, through the inhibition of nitric oxide and cyclooxygenase-2 production [28].

Given these premises and the existence of conflicting information, it is important to evaluate the effects of these two compounds in animal models to ensure their safety use when introduced into food products, such as for the preservation and storage of cereals and legumes. For this, female mice of the FVB/n inbred strain were use. This strain presents a low incidence of hepatic lesions and spontaneous tumors when compared with other strains [57], ensuring that all liver changes observed in the assay are derived from compound administration and not from the strain’s genetic background.

During the experimental protocol, humane endpoints were evaluated to ensure the animals’ welfare and detect changes induced by the procedures early. A mortality index of 20% was observed in the acute toxicity group (group VII), probably due to hemorrhagic enteritis (macroscopically identified, but not confirmed by histopathology). The mortality index was lower when compared with that observed in an acute toxicity study developed by NTP [58], in which all animals receiving 10% of eugenol and bis-eugenol in their diet (corresponding to 100 mg/kg) died before the end of the experimental protocol, lasting 14 days. The results of humane endpoints indicate that the ingestion of high doses of eugenol and pulegone promotes changes in several physiological parameters, as shown by the elevated number of changes observed in the acute toxicity group. An increase in the eugenol and pulegone doses (from groups II to IV) led to changes in the animals’ behavior and welfare, namely in their hair appearance and grooming. Immediately after the beginning of the ingestion of a diet supplemented with pulegone, animals from group VI presented changes in the color of the feces, which was reverted in the following weeks. In the consulted bibliography, there are no reports observing or analyzing the feces of animals treated with pulegone; as such, in the near future, it would be interesting to evaluate this parameter and clarify the mechanisms underlying this change.

The animals’ body weight increased between the beginning and the end of the experimental protocol. A marked decrease was observed in the body weight of animals from the acute toxicity group (group VII) between the beginning and the end of the experimental protocol. At the end of the experiment, the body weight of animals from group VII was lower when compared with the control group (group I), which may be related to the incorporation of high quantities of eugenol and pulegone in the diet. The groups IV and VII presented a negative PWG. The animals from group IV were chronically fed with a diet supplemented with both eugenol and pulegone in higher doses (7.8 mg of each compound), when compared with groups II (2.6 mg eugenol + 2.6 mg pulegone) and III (5.2 mg eugenol + 5.2 mg pulegone). The diets of groups V (7.8 mg eugenol) and VI (7.8 mg pulegone) were supplemented with the compounds, separately, suggesting that they can interact with each other and affect the animals’ body weight as observed in group IV. Body weight decrease is an indicator of toxicity, and a marked decrease of animals’ body weight was observed in the acute toxicity group (group VII), possibly because of the large quantities of eugenol and pulegone incorporated in the diet. Body weight loss was previously reported in B6C3F1 mice receiving 2500, 25,000, or 50,000 mg/kg of eugenol in their diet for 14 days [58]. In the same series of studies, mice receiving diets with 0, 400, 800, 1500, 3000, or 6000 mg/kg of eugenol for 91 days did not present significant changes in body weight among groups (*p* > 0.05). As far as we have learned from the literature search, studies evaluating the incorporation of pulegone into rodents’ diet have not been performed.

The mean liver weight was higher in group III (5.2 mg eugenol + 5.2 mg pulegone) when compared with groups II (2.6 mg eugenol + 2.6 mg pulegone) and IV (7.8 mg eugenol + 7.8 mg pulegone), suggesting that the liver weight tends to increase with the exposure to 5.2 mg/day of eugenol and pulegone, possibly because the metabolization of these compounds may occur mainly in this organ. However, the liver weight of group VII (1000 mg eugenol + 1000 mg pulegone) was lower when compared with the control group (group I), which may be related to the toxicity of these compounds in high doses. No justifications have been found in the literature for the differences in the spleen and lung relative weight between groups. In a study previously performed by NTP [59], mice receiving 150 and 300 mg/kg of pulegone by gavage presented higher relative liver weight when compared with the control group. In the present trial, the chronic toxicity groups (groups II to VI) presented a higher relative liver weight when compared with the control group (group I). The groups II, III, and IV supplemented with increasing doses of eugenol presented a similar relative liver weight that is difficult to understand. Dose-related increases in liver weight are commonly observed in repeat-dose toxicity studies performed in rodents [60].

The hematocrit was similar among groups. NTP [59] demonstrated that the supplementation of rats with 9.38 to 150 mg/kg of pulegone by gavage decreased the hematocrit value. Our results are not in agreement with those presented by NTP; however, it is important to note that there are differences between the two protocols, namely the administration route, the dose, and the species used. Kumar et al. [61] observed an increase in albumin serum levels of Wistar rats administered with eugenol (3 mg/kg) by gavage for 21 days, which is not in accordance with that observed in our work. Previous studies reported an increase in the serum levels of cholesterol associated with the administration of pulegone [62]. Although an increase in the levels of cholesterol was observed in groups IV (7.8 mg eugenol + 7.8 mg pulegone) and VI (7.8 mg pulegone), these differences did not reach the level of statistical significance between the groups (*p* > 0.05). Inversely, other studies reported a decrease in the serum levels of cholesterol associated with the administration of eugenol [61], which is not in agreement with our results. Markakis et al. [61] observed a decrease in the urea serum levels of Wistar rats treated with eugenol (1.5 mg/mL) by gavage. Accordingly, a decrease in the urea serum levels was observed in the group treated with eugenol (group V, 7.8 mg), when compared with the remaining groups. According to Harb et al. [63], the Wistar rats treated with eugenol at a concentration of 10 and 100 mg/kg, by gavage, presented lower serum levels of triglycerides. Moreover, a decrease in the triglycerides levels was also observed in Wistar rats and rabbits administered with pulegone [62,64]. In our trial, a reduction in the triglycerides levels was observed in the group of the chronic toxicity assay treated with a higher dose of eugenol in combination with pulegone (group IV, 7.8 mg eugenol + 7.8 mg pulegone) and in the acute toxicity group (group VII, 1000 mg eugenol + 1000 mg pulegone). A decrease in alanine aminotransferase serum levels was also reported in rats receiving the extract of the *Ziziphora tenuior* plant, containing a high content of pulegone. Inversely, a rise in the serum levels of this enzyme was observed in rats to which eugenol was given orally in different doses over a 15-day period [65,66]. Accordingly, a rise in the serum levels of alanine aminotransferase was observed in the acute toxicity group (group VII). The non-existence of dose-related changes in the serum parameters of our animals, and the differences with the studies previously performed, in terms of strain, dose, route, and duration of administration, make the discussion of these results difficult.

Escobar et al. [67] assessed the genotoxicity of piperine essential oil (containing 64.7% of pulegone) in Wistar rats’ lymphocytes, and no evidence of DNA damage was found. Yogalakshmi et al. [68] observed that pretreatment with eugenol decreased DNA damage caused by thioacetamide. Moreover, Tiku et al. [69] demonstrated that eugenol has radioprotective potential in mice exposed to gamma radiation. In our trial, the genotoxicity of eugenol and pulegone was evaluated through the comet assay in the blood samples. Group IV (treated with the combination of 7.8 mg eugenol + 7.8 mg pulegone) presented the highest GDI, and it was statistically different from groups I (control) and III (5.2 mg eugenol + 5.2 mg pulegone). These results seem to indicate that eugenol and pulegone, when administered together in a dose of 7.8 mg, cause DNA damage. Although no significant differences were observed among the remaining groups, it is worth noting that the acute toxicity group (group VII) does not appear to present signs of genotoxicity, despite been treated with higher doses of eugenol and pulegone when compared with group IV. No studies were found in the literature with same experimental conditions or groups (combined administration of eugenol and pulegone, doses administered), so it is not possible to compare or draw an exact conclusion from these results. It is also important to note that the comet assay is not able to detect DNA fragments resulting from necrosis or apoptosis, so in a future study of chronic or acute toxicity it would be interesting to investigate the genetic damage caused by these two processes [70,71]. As the stomach is the first organ exposed in oral administration, it would be also important to assess the genetic damage extension in the cells of stomach mucosa [72].

The activities of antioxidant enzymes SOD, CAT, GR, and GST were measured in the animals’ livers. The results obtained in the present trial are difficult to explain, since there are no studies where the eugenol and pulegone are evaluated together, making it impossible to understand their synergistic or antagonistic potential. According to Nejad et al. [73], small changes in the molecular structure of these compounds may lead to different molecular properties and biological activity. Reddy and Lokesh [74] concluded that eugenol has high antioxidant and anti-inflammatory potential, increasing the SOD, CAT, and GST activity in rats fed with coconut oil. Ma et al. [75] also verified that eugenol reverted the oxidate stress in rats with spinal cord injury by increasing the SOD and CAT activity. Garabadu et al. [76] tested the protective effects of eugenol in stress-induced irritable bowel syndrome in rats and observed an increased activity of SOD in certain regions of the brain of animals treated with this compound. Huang and colleagues [77] verified that eugenol inhibited an inflammatory response in acute lung lesions and increased SOD, CAT, and GST activities. There are many studies suggesting that eugenol has high protective activity against free radicals’ formation, with Kaur et al. [78], Abd El Motteleb et al. [79], and Mnafgui et al. [80] showing that eugenol increases the activity of the antioxidant enzymes mentioned above. The pulegone toxicity is largely attributed to its metabolism to reactive species, as stated above. These metabolites bind to macromolecules and decrease glutathione levels, leading to toxicity. Changes in the liver gene expression of rats treated with an oral administration of pulegone (400 mg/kg) suggested an activation of Nrf2, which is a common response to oxidative stress and GSH depletion [81]. There are few studies evaluating the toxicological effects of pulegone in animal models and its relation with antioxidant enzymes, thus hampering the results discussion. The SOD activity was not different among the groups. However, according to the literature, the group V should present increased SOD levels, which was not observed in the present study. This group showed higher CAT activity when compared with the control group, as previously reported by other researchers. Eugenol has a high ability to scavenge free radicals, and the increased CAT activity suggests an increase in the H_2_O_2_ degradation and, consequently, a decrease in the potential toxicity of the cell [81]. An increase was also observed in the CAT activity in groups III and IV, when compared with the control group (group I). The highest GR activity was observed in the acute toxicity group (group VII) treated with the highest doses of eugenol and pulegone in combination. These results are in accordance with those obtained by Al-Trad et al. [81], who observed increased GR activity in rats with rheumatoid arthritis treated with eugenol. This increase in GR activity means that more reduced glutathione (GSH) is being recycled, and therefore the GSH/GSSG (oxidized glutathione) ratio is also increased. These data are not in accordance with those already known for pulegone, which have suggested that it is a depletory of glutathione levels. High GSH levels may also be linked to liver metastasis and melanoma [81], which was not observed in our animals. Group VI presented a higher GST activity when compared with group V and the control. These data are in accordance with Rabinovich et al. [82], who observed increased GST activity in rats with carbon tetrachloride-induced oxidative stress treated with pulegone. Group V also presented higher GST activity when compared with control animals, as previously demonstrated by Adefegha et al. [83], and Vidhya and Devaraj [84]. The non-existence of dose-related changes in the activity of antioxidant enzymes make the discussion of results challenging.

None of the animals presented significant histological changes in the heart and spleen. Inflammatory infiltrates were observed in the liver, kidneys, and lung. The evidence of carcinogenicity was equivocal in a study developed by NTP, where increasing doses of eugenol were administered in the diet of mice for two weeks [58]. Later, in 2011, the NTP observed an increased incidence of both neoplastic and non-neoplastic lesions, such as centrilobular hepatocellular hypertrophy, in mice supplemented with pulegone for two years [59]. In the present study, all animals from the acute toxicity group (group VII) presented marked centrilobular hepatocellular hypertrophy, which may be responsible for the increased liver weight, and 40% presented hepatocyte apoptosis, which confirms the liver toxicity. Previous studies pose the possibility that eugenol and pulegone are bioavailable in the lung. In the present study, significant differences regarding the inflammatory infiltrate score were only observed in the lung. However, there are some experimental protocols studying the beneficial and protective effects of eugenol in damaged lungs [19,77,85,86].

## 5. Conclusions

Eugenol and pulegone affected some physiological parameters, namely the general health status of the animals, food and water consumption, PWG, and the relative weight of the lung, spleen, and liver. These compounds did not affect the hematocrit value but modulated inflammatory infiltrate in the lung. Both compounds induced DNA damage. When administered in high doses in combination, eugenol and pulegone increased the GR enzyme. Eugenol and pulegone may exert negative effects when incorporated into the diet of FVB/n mice, depending on the dose, and whether the application is separate or in combination. However, the extent of these effects is not fully understood, and the administration of the compounds combined in different doses did not provide a precise perception of the results. In future work, it would be interesting to perform an experimental study with different doses of eugenol and pulegone, administered separately and in combination, to evaluate the beneficial or harmful effects of each one in the animals, as well as to understand the synergistic or antagonistic action between them.

## Figures and Tables

**Table 1:** Humane endpoints applied in the experimental protocol. Adapted from [41,42,43].

Parameter	Score			
	0	1	2	3
Body condition	Normal	Changed body condition	Emaciated	---
Body weight		Weight loss of <10%	Weight loss of 10–20%	Weight loss of >20%
Posture	Normal	Curved	---	---
Hair appearance and grooming	Normal	Lack of grooming	Bad hair condition, chromodachryorrhea	Hair in very bad condition, severe chromodachryorrhea
Mucosa	Normal	Slightly anemic	Moderately anemic	Severely anemic (Euthanasia)
Eyes, ears, and whiskers	Normal	Eyes partially closed, dropped ears, whiskers flattened and elongated to the tip of the nose	Eyes completely closed, dropped ears, whiskers flattened and elongated to the tip of the nose	---
Mental status	Normal	Inactive, stereotyped behavior	Stupor	Coma (Euthanasia)
Response to external stimuli	Normal	Moderate	Moderate with vocalization	Violent
Hydration status (skin pinch)	Normal	Abnormal skin pinch test (>2 s)	---	---
Respiratory rate	Normal	Increased	Decreased	Abdominal breathing
Heart rate	Normal	Increased	Decreased	----
Stool appearance	Normal	Diarrhea, changed color	Black (with digested blood)	With blood in nature (Euthanasia)
Convulsions	Absence	Presence	---	---

**Table 2:** Score of humane endpoints for each group during the experimental protocol.

Group (*n* = 5)	Mean Score of Humane Endpoints			
	Week 1	Week 2	Week 3	Week 4
I (control)	0	0	0	0
II (2.6 mg eugenol + 2.6 mg pulegone)	0	0.4	0.8	0.8
III (5.2 mg eugenol + 5.2 mg pulegone)	0	0	0	0
IV (7.8 mg eugenol + 7.8 mg pulegone)	1	0.6	0.2	1
V (7.8 mg eugenol)	0	0	0	0
VI (7.8. mg pulegone)	0	0.2	0.2	0.6
VII (1000 mg eugenol + 1000 mg pulegone)	0	0	0	6.8

**Table 3:** Number and percentage of animals from all groups with changed parameters throughout the experimental protocol.

Changed Parameter	Score	Week of the Protocol			
		1	2	3	4
Body condition	1	---	---	---	Group VII (*n* = 4; 80%)
Body weight	2	Group IV (*n* = 1; 20%)	Group IV (*n* = 1; 20%)	Group II (*n* = 1; 20%)	---
	3	Group IV (*n* = 1; 20%)	---	---	Group VII (*n* = 5; 100%)
Posture	1	---	Group IV (*n* = 1; 20%) Group VI (*n* = 1; 20%)	Group IV (*n* = 1; 20%) Group VI (*n* = 1; 20%)	Group VII (*n* = 4; 80%)
Hair appearance and grooming	1	---	---	---	Group IV (*n* = 5; 100%) Group VI (*n* = 3; 60%) Group VII (*n* = 4; 80%)
Eyes, ears, and whiskers	1	---	---	---	Group VII (*n* = 1; 20%)
Mental status	1	---	Group II (*n* = 2; 40%)	Group II (*n* = 2; 40%)	Group II (*n* = 4; 80%) Group VII (*n* = 3; 60%)
	3	---	---	---	Group VII (*n* = 1; 20%)

**Table 4:** Initial and final food and water consumption per animal for all experimental groups (mean).

Group (*n* = 5)	Food Consumption (g)		Water Consumption (g)	
	Initial	Final	Initial	Final
I (control)	5.70	4.60	5.36	4.33
II (2.6 mg eugenol + 2.6 mg pulegone)	5.77	5.17	5.90	4.97
III (5.2 mg eugenol + 5.2 mg pulegone)	4.40	4.39	6.24	4.95
IV (7.8 mg eugenol + 7.8 mg pulegone)	3.09	3.31	9.89	7.11
V (7.8 mg eugenol)	5.62	4.76	5.72	5.54
VI (7.8. mg pulegone)	3.93	3.75	6.67	5.43
VII (1000 mg eugenol + 1000 mg pulegone)	4.44	0.40	5.61	1.39

**Table 5:** Initial and final body weight (g) per animal (mean ± SE), and ponderal weight gain (PWG; %) for all experimental groups.

Group (*n* = 5)	Body Weight (g)		PWG (%)
	Initial	Final	
I (control)	25.83 ± 1.09	29.51 ± 1.95 ^a^	15.05 ^b^
II (2.6 mg eugenol + 2.6 mg pulegone)	24.99 ± 2.21	26.89 ± 2.80	11.19
III (5.2 mg eugenol + 5.2 mg pulegone)	26.33 ± 2.32	28.42 ± 1.92	10.73
IV (7.8 mg eugenol + 7.8 mg pulegone)	26.89 ± 1.59	25.66 ± 1.42	−0.88 ^c^
V (7.8 mg eugenol)	26.27 ± 1.54	27.73 ± 1.50	9.04
VI (7.8. mg pulegone)	24.99 ± 1.73	26.59 ± 1.32	5.56
VII (1000 mg eugenol + 1000 mg pulegone)	28.00 ± 1.02	18.18 ± 1.45	−48.77

^a^ Statistically different from group VII (*p* = 0.000); ^b^ Statistically different from groups IV (*p* = 0.003) and VII (*p* = 0.000); ^c^ Statistically different from groups II (*p* = 0.035) and III (*p* = 0.047).

**Table 6:** Organs’ weight (g) (mean ± SE) in all experimental groups.

Group (*n* = 5)	Organs’ Weight (g)					
	Heart	Lung	Right Kidney	Left Kidney	Spleen	Liver
I (control)	0.144 ± 0.029	0.206 ± 0.026	0.192 ± 0.018	0.198 ± 0.011	0.128 ± 0.019	1.432 ± 0.089
II (2.6 mg eugenol + 2.6 mg pulegone)	0.134 ± 0.015	0.182 ± 0.026	0.178 ± 0.028	0.178 ± 0.025	0.140 ± 0.02	1.384 ± 0.199
III (5.2 mg eugenol + 5.2 mg pulegone)	0.128 ± 0.029	0.184 ± 0.022	0.170 ± 0.014	0.174 ± 0.015	0.174 ± 0.015	1.546 ± 0.083 ^a^
IV (7.8 mg eugenol + 7.8 mg pulegone)	0.112 ± 0.004	0.188 ± 0.011	0.144 ± 0.027	0.158 ± 0.016	0.082 ± 0.016	1.418 ± 0.082
V (7.8 mg eugenol)	0.148 ± 0.022	0.214 ± 0.030	0.178 ± 0.008	0.176 ± 0.037	0.134 ± 0.021	1.432 ± 0.094
VI (7.8. mg pulegone)	0.120 ± 0.016	0.190 ± 0.012	0.154 ± 0.009	0.152 ± 0.008	0.110 ± 0.020	1.430 ± 0.080
VII (1000 mg eugenol + 1000 mg pulegone)	0.100 ± 0.016	0.188 ± 0.024	0.133 ± 0.013	0.133 ± 0.010	0.060 ± 0.018	0.848 ± 0.097 ^b^

^a^ Statistically different from groups I (*p* = 0.006), II (*p* = 0.000) and IV (*p* = 0.001); ^b^ Statistically different from group I (*p* = 0.000).

**Table 7:** Number and percentage of animals with inflammatory infiltrate and necrosis, and respective score (mean ± SE).

Group (*n* = 5)	Score	Number of Animals (%)			
		Inflammatory Infiltrate			Necrosis/Apoptosis
		Liver	Kidneys	Lung	Liver
I (control)	0	4 (80%)	4 (80%)	3 (60%)	5 (100%)
	1	1 (20%)	1 (20%)	2 (40%)	---
	2	---	---	---	---
Mean ± SE		0.20 ± 0.45	0.20 ± 0.45	0.40 ± 0.55 ^a^	0.00 ± 0.00
II (2.6 mg eugenol + 2.6 mg pulegone)	0	2 (40%)	4 (80%)	4 (80%)	5 (100%)
	1	3 (60%)	1 (20%)	1 (20%)	---
	2	---	---	---	---
Mean ± SE		0.60 ± 0.55	0.20 ± 0.45	0.20 ± 0.45 ^a^	0.00 ± 0.00
III (5.2 mg eugenol + 5.2 mg pulegone)	0	2 (40%)	2 (40%)	3 (60%)	5 (100%)
	1	3 (60%)	2 (40%)	2 (40%)	---
	2	---	1 (20%)	---	---
Mean ± SE		0.60 ± 0.55	0.80 ± 0.84	0.40 ± 0.55 ^a^	0.00 ± 0.00
IV (7.8 mg eugenol + 7.8 mg pulegone)	0	2 (40%)	1 (20%)	---	5 (100%)
	1	2 (40%)	3 (60%)	1 (20%)	---
	2	1 (20%)	1 (20%)	4 (80%)	---
Mean ± SE		0.80 ± 0.84	1.00 ± 0.71	1.80 ± 0.45	0.00 ± 0.00
V (7.8 mg eugenol)	0	---	1 (20%)	---	5 (100%)
	1	5 (100%)	3 (60%)	3 (60%)	---
	2	---	1 (20%)	2 (40%)	---
Mean ± SE		1.00 ± 0.00	1.00 ± 0.71	1.40 ± 0.55	0.00 ± 0.00
VI (7.8. mg pulegone)	0	1 (20%)	1 (20%)	1 (20%)	5 (100%)
	1	4 (80%)	4 (80%)	4 (80%)	---
	2	---	---	---	---
Mean ± SE		0.80 ± 0.45	0.80 ± 0.45	0.80 ± 0.45	0.00 ± 0.00
VII (1000 mg eugenol + 1000 mg pulegone)	0	1 (20%)	4 (80%)	1 (20%)	3 (60%)
	1	4 (80%)	1 (20%)	4 (80%)	2 (40%)
	2	---	---	---	^---^
Mean ± SE		0.80 ± 0.45	0.20 ± 0.45	0.80 ± 0.45	0.40 ± 0.55

^a^ Statistically different from groups IV and V (*p* < 0.05).

**Table 8:** Number and percentage of animals with centrilobular hepatocellular hypertrophy and hepatocyte karyomegaly.

Group (*n* = 5)	Number of Animals (%)		
	Centrilobular Hepatocelular Hypertrophy	Marked Centrilobular Hepatocelular Hypertrophy	Hepatocyte Karyomegaly
I (control)	3 (60%)	---	3 (60%)
II (2.6 mg eugenol + 2.6 mg pulegone)	3 (60%)	---	3 (60%)
III (5.2 mg eugenol + 5.2 mg pulegone)	4 (80%)	---	4 (80%)
IV (7.8 mg eugenol + 7.8 mg pulegone)	4 (80%)	---	4 (80%)
V (7.8 mg eugenol)	4 (80%)	---	4 (80%)
VI (7.8. mg pulegone)	3 (60%)	---	3 (60%)
VII (1000 mg eugenol + 1000 mg pulegone)	---	5 (100%)	5 (100%)

## Data Availability

The data presented in this study are available on request from the corresponding author.
